# Acute effects of intranasal ocytocin on affective empathy of patients with refractory schizophrenia and healthy controls: results of a randomized clinical trial

**DOI:** 10.1192/j.eurpsy.2024.1590

**Published:** 2024-08-27

**Authors:** F. D. L. Osório, A. C. Ferreira

**Affiliations:** São Paulo University, Ribeirão Preto, Brazil

## Abstract

**Introduction:**

Oxytocin (OXT) is a neuropeptide associated with social behavior and the modulation of neural circuits related to social cognition and emotion regulation. Schizophrenia is a mental disorder that causes impairment in different areas of social cognition, including empathy. A systematic review of the literature showed positive effects of exogenous administration of this hormone on the empathy of individuals without psychopathology, especially in the affective domain. Studies on the effect of OXT on empathy in patients with schizophrenia are very limited, being restricted to the cognitive domain.ations must be overcome in future studies. The effects associated with chronic use of the hormone should be the subject of future studies.

**Objectives:**

to evaluate the effect of a single dose of intranasal OXT (24UI) on affective empathy in individuals with refractory schizophrenia and healthy controls.

**Methods:**

a double-blind, randomized, placebo-controlled clinical trial was conducted. A convenience sample of 51 adult men (mean age 34.4 ± 7.6, >10 years of education) was recruited, 20 of whom were diagnosed with refractory schizophrenia according to the DSM-5 (exclusively using clozapine or clozapine + mood stabilizer and/or benzodiazepine) and 31 healthy controls. They were randomized into four groups and received OXT or placebo (PLA – vehicle: SCH-OXT (N=11), SHC-PLA (N=9), HC-OXT (N=15), HC-PLA (N= 16)). Before and after 50 minutes of administering the substance, they performed an affective empathy task (Multifaceted Emphaty Test – MET).

**Results:**

the baseline levels of affective empathy of patients with schizophrenia were lower compared to healthy controls when faced with negative stimuli (p=0.003), but not positive ones (p=0.39). After the administration of OXT and PLA (post-pre), a small increase in empathy levels was observed in all groups, which did not reach statistical significance (positive stimuli: ΔSCH-OXT = 0.16±1.08; ΔSHC-PLA= 0.53±1.44, ΔHC-OXT= 0.02±0.67, ΔHC-PLA= 0.24±0.45, p=0.85; negative stimuli: ΔSCH-OXT = 0.20±1.31; ΔSHC-PLA= 1.16±0.79, ΔHC-OXT= 0.12±0.99, ΔHC-PLA= 0.31±0.57, p=0.11).

**Conclusions:**

the acute effects of intranasal OXT did not favor improvements in the levels of affective empathy, either in patients with schizophrenia or in healthy controls, contrary to the hypotheses of this study. The limited sample size and context-dependent aspects of OXT may explain these findings. These methodological limitations must be overcome in future studies. The effects associated with chronic use of the hormone should be the subject of future studies.

**Disclosure of Interest:**

None Declared

